# Sociology of Low Expectations: Recalibration as Innovation Work in Biomedicine

**DOI:** 10.1177/0162243915585579

**Published:** 2015-11

**Authors:** John Gardner, Gabrielle Samuel, Clare Williams

**Affiliations:** 1 University of York, York, UK; 2 Brunel University, Uxbridge, UK

**Keywords:** expertise, futures, translational medicine, neurotechnology

## Abstract

Social scientists have drawn attention to the role of hype and optimistic visions of the future in providing momentum to biomedical innovation projects by encouraging innovation alliances. In this article, we show how less optimistic, uncertain, and modest visions of the future can also provide innovation projects with momentum. Scholars have highlighted the need for clinicians to carefully manage the expectations of their prospective patients. Using the example of a pioneering clinical team providing deep brain stimulation to children and young people with movement disorders, we show how clinicians confront this requirement by drawing on their professional knowledge and clinical expertise to construct visions of the future with their prospective patients; visions which are personalized, modest, and tainted with uncertainty. We refer to this vision-constructing work as recalibration, and we argue that recalibration enables clinicians to manage the tension between the highly optimistic and hyped visions of the future that surround novel biomedical interventions, and the exigencies of delivering those interventions in a clinical setting. Drawing on work from science and technology studies, we suggest that recalibration enrolls patients in an innovation alliance by creating a shared understanding of how the “effectiveness” of an innovation shall be judged.

## Introduction

Deep brain stimulation (DBS) is a therapeutic technique that involves using a pacemaker-like device to deliver constant, carefully targeted electrical stimulation to specific areas of the brain. It was first approved as a treatment for managing some of the symptoms of Parkinson’s (PD) in the late 1990s, and subsequently it has been used to treat tens of thousands of individuals worldwide. Its success has led clinicians and device manufacturers to explore other applications for the DBS technique. In 2003, it was approved as means for managing dystonia, a movement disorder characterized by sustained and intermittent involuntary muscular contraction, which can cause painful, crippling body postures. Although it is not a cure, being reserved for people who no longer respond to more conventional treatments, DBS has been heralded as a life-changing medical therapy for people with PD or dystonia (Chou, Grube, and Patil 2011). Many of those individuals treated with DBS have experienced notable improvements in functionality, and some individuals have experienced dramatic improvements. Reports of previously housebound individuals with debilitating symptoms subsequently gaining independence are not uncommon. Proponents argue that, unlike the ablative therapies it has supplanted, DBS does not cause irreversible damage to the brain (Ardouin et al. 1999). And while the initial cost of DBS is high, there is emerging evidence to suggest that it may be, in the long-term, a cost-effective treatment (Fraix et al. 2006). Indeed, DBS has become a mainstream treatment in many countries for people with PD with symptoms that no longer respond to medications (Talan 2009). There is considerable hope among clinicians and device manufacturers that DBS will prove to be a clinically effective and cost-effective intervention not only for dystonia but also for other neurological and psychiatric disorders including obsessive compulsive disorder (Greenberg et al. 2006) and depression (Mayberg et al. 2005).

Not surprisingly, DBS has been presented as a source of hope for prospective patients, and like many novel biomedical interventions DBS has become the subject of hype. Much of the media coverage of DBS, for example, has involved “over-optimistic portrayals,” focusing on individual cases that do remarkably well while ignoring the more subtle improvements experienced by the majority of patients. Such positive media portrayals often draw upon the hopeful, positive representations produced by scientist and innovators themselves (Gilbert and Ovadia 2011). Indeed, as Schlaepfer and Fins (2010) have illustrated, single-case studies of DBS within the scientific literature have highlighted positive secondary effects, even when the primary goals of the research have not been achieved. Commentators have argued that by emphasizing positive effects and perpetuating over-optimistic portrayals, such coverage is generating unrealistic expectations among prospective patients, many of whom have debilitating neurological disorders and are desperate for some form of reprieve (Racine et al. 2007).

Social scientists have noted that this hype and the over-optimistic visions of future are an integral part of the innovation process. In effect, they provide momentum to innovation projects by attracting resources and encouraging necessary alliances (Brown, Rappert, and Webster 2000). Yet these over-optimistic constructions of the future can create challenges for clinicians who wish to provide novel therapies to patients. As other researchers have noted, clinicians must attain informed consent from patients, but this cannot be achieved if patients maintain unrealistic expectations of what the intervention can do for them (Jox et al. 2012). Additionally, if clinical outcomes consistently fail to match patients’ expectations, the reputation of clinicians and the intervention itself can suffer.

There is, then, something of a tension between hyped visions of the future that tend to circulate in some domains and provide momentum to innovation projects, and the exigencies of integrating a novel technology into an innovative therapy within a responsible clinical service. In this article, we draw attention to this tension as it relates to DBS. In particular, we explore the activities of a multidisciplinary team of clinicians providing DBS to children and young people with dystonia. The team, which we refer to as the pediatric motor disorder service (PMDS), is based at a children’s hospital in the United Kingdom and is one of a few teams worldwide that provide DBS specifically to children with a severe neurological disorder. As we will illustrate, the clinicians feel that it is not unusual for children and families attending the service to have unrealistic visions about what DBS can offer them. Using the PMDS as a case study, we argue that clinicians working at the “coal-face” of innovation engage in a particular kind of work with patients, which we refer to as *recalibration*. *Recalibration* involves co-constructing a vision of the future with each patient, and encouraging the patient to adopt that vision and thus recalibrate their expectations with it. Drawing on the “sociology of expectations” literature (and the emerging “sociology of low expectations” literature), we suggest that *recalibration* (and the modest, highly personalized, and uncertain futures that this work produces) is an important and as yet unacknowledged aspect of translational medicine.

## Innovation and Expectation

Various studies under the banner of the “sociology of expectations” have explored the way in which future-orientated discourses drive and shape innovation projects (van Lente and Rip 1998; Borup et al. 2006; Brown, Rappert, and Webster 2000; Brown and Michael 2003; Kitzinger and Williams 2005). Much of this work has explored the construction, dissemination, and effects of hype and optimistic visions of the future, in which the innovation and those individuals and institutions involved are presented in a highly favorable frame. Such visions, it is argued, play a performative role in innovation projects: optimistic, future-orientated rhetoric animates innovation projects by encouraging the building of alliances (Borup et al. 2006; Brown, Rappert, and Webster 2000). By deploying visions of the future (which often involve narratives of “breakthrough” and “discovery”) and generating promissory expectations, institutions can enroll a potentially diverse array of allies into a common innovation project. Real-time representations of the future also have structuring effects on innovation alliances: they delineate and coordinate institutional and professional roles, and by envisaging particular beneficial outcomes and payoffs, they prescribe responsibilities to those involved.

The “sociology of expectations” literature, then, has demonstrated that examining the construction and dissemination of such futures provides important insights into the dynamics of innovation and social and technical change, particularly in biomedicine (Hedgecoe and Martin 2003). While much of the work in this area has focused on “optimistic” future abstractions (or hype), a small, nascent body of work has drawn attention to the less-promissory visions of the future that accompany biomedical innovation projects. This work has examined the nature of “low expectations” and what role they might play in innovation projects as a whole. Tutton, for example, focuses on the biotech industry in the United States that, in order to attract potential investors and resources, produces and circulates highly optimistic forward-looking statements. Biotech firms are permitted to do this by the US Securities and Exchange Commission (SEC) on the condition that they identify and stipulate the possible risk factors that would prevent such a future from materializing. They must, in other words, carefully consider and describe all the things that could possible go wrong, and by doing this, they can protect themselves against accusations of misleading investors and the public. These pessimistic projections are filed with the SEC, and as Tutton notes, they employ particular words and phrasing, such as “uncertainty,” “we cannot predict,” and “may also have adverse effect.” Tutton suggests that these projections are not simply a matter of accountability. Rather, they should be seen as part of an “anticipatory regime,” in which companies are “forced to tack back and forth between pessimistic and optimistic forecasts of equally conditional futures” (Tutton 2011, 419). Such pessimistic projections are not performative (firms wish to avoid them rather than enact them), but they are nonetheless an important part of the work that must be done in order to provide momentum to a biomedical innovation project.

Other scholars have noted a similar intertwining of high and low expectations among the scientists and researchers involved in biomedical research. Pickersgill, for example, explored the perspectives of neuroscientists and clinicians on neuroscientific research in mental health and personality disorders (Pickersgill 2011). Such research has attracted considerable investment and is the subject of much optimism, and to some extent this optimism was shared by scientists and clinicians working within the field. However, Pickersgill’s respondents expressed notable ambivalence about the potential clinical impact of neuroscience and were skeptical of the more promissory neuroscience claims. Indeed, some suggested that such positive coverage might actually be detrimental to the field. In large part, the ambivalent views of clinicians were derived from their clinical understanding of personality disorders and their impression that some neuroscientific claims were simply not clinically useful. In a similar vein, Fitzgerald has explored the perspectives of scientists working in neurobiological autism research (Fitzgerald 2014). Again, the scientists interviewed mirrored some of the hope and positive anticipation that was associated with autism research more generally. Some, for example, were excited by the prospect that technological advancements would shed light on the workings of the brain and provide new insights into neurological and psychiatric pathology. Yet scientists also expressed disappointment in these “wonderful new technologies” and unease about the speculative hype surrounding them. They foresaw a complex future in which the useful contribution of neuroscience autism research was uncertain: indeed, they expressed low expectations of the future. As with Pickersgill’s respondents, these scientists talked about their field in what Fitzgerald refers to as “entangled registers of both promising hope and deflated uncertainty” (Fitzgerald 2014, 241). Fitzgerald suggests that this ambivalence represents an intellectual language that enables scientists to navigate and work within the “intermediate terrain” of an evolving biomedical field characterized by unknowns (such as the unknown etiology of autism). In other words, an innovation project may fail to progress toward a clearly envisaged, optimistic future, but this does mean that it stops dead: it is nonetheless propelled toward *a* future by scientists and researchers who are both cautiously hopeful and constructively skeptical.

This nascent “sociology of low expectations” scholarship illustrates that biomedical innovation projects may not simply be animated by high expectations alone. Rather, the dynamism of innovation emerges from a complex intertwining of low and high expectations; an interplay of promise, hope, and optimism, and uncertainty, pessimism, and ambivalence. As Fitzgerald puts it in regard to neuroscientific research, negative expectations are “not only thickly present; they may actually be important for the maintenance of some particularly ambiguous neuroscience projects” (Fitzgerald 2014, 242). Indeed, this insight aligns with Moreira and Palladino’s (2005) argument that contemporary biomedicine is shaped by two logics: the “regime of hope” and the “regime of truth.” The former is characterized by the optimistic perception that research activities are warranted by the promise of a high-reward payoff such as a miraculous cure. The latter, on the other hand, is characterized by “the investment in what is positively known, rather than what can be,” and the belief that “most medical therapies are less effective than claimed” (p. 67). Biomedical endeavors are constituted by aggregates and modes of organizing that follow either or both of these logics: prospective patients, for example, are rallied by hope, while regulators and patient support groups may be rallied by “truth.” Tutton suggests that biotech companies move back and forth between the two regimes, and the work of Fitzgerald and Pickersgill suggests that researchers and clinicians can occupy some sort of intermediate position by drawing on understandings from both.

In this article, we also draw attention to the complex dynamics of hope, uncertainty, promise, and doubt that animate innovative biomedical activities. However, unlike previous work in the “sociology of (low) expectations” which has tended to focus on the constructed futures that animate the early stages of research or innovation projects, this article explores the future-constructing work of pioneering clinicians as they work with patients at the adoption stage of innovation. The development and dissemination of DBS has certainly been associated with highly optimistic forward-looking statements, but using the PMDS as a case study, we illustrate that dissemination and incorporation into clinical services also involves the careful construction of visions of the future that are uncertain and less optimistic. The PMDS clinicians carefully construct personalized, modest, and uncertain visions of the future with their patients; a form of clinical labor that we refer to as *recalibration*. Recalibration involves, we argue, enrolling patients and their families in a *regime of truth*, in which the likely benefits of DBS and its limitations, are rendered explicit. Consequently, patients and families are prompted to rationally reflect on their orientation toward the future, and they are encouraged to re-orientate themselves toward an expected, mandated future based upon diagnostic truth. This is not to say that patients and their families are disengaged from the regime of hope: rather, hope for a dramatic recovery remains a prominent undercurrent.

The premise of this article is that *recalibration* is an important component of the innovation process, specifically in the clinical sites where novel biomedical projects are being translated in clinical therapies. While DBS for PD has been widely adopted, there is still considerable uncertainty surrounding its future as a treatment for other disorders such as dystonia. Indeed, the Nuffield Council on Bioethics recent report *Neurotechnologies: Intervening in the Brain* (2013) identified DBS as one of several highly promising neurotechnologies for which there is “a great need,” but which are also surrounded by “great uncertainty.” The report suggests that DBS occupies a tenuous position between experimental therapy and routine clinical treatment. The clinicians who work in this terrain can be said to be clinical pioneers: the degree to which the benefits of novel techniques such as DBS are realized depends on their capacity to integrate and adapt the technology into day-to-day clinical services involving patients (Hopkins et al. 2007; Morlacchi and Nelson 2011). Recalibration is a vital part of this process: as we demonstrate, it enables clinicians to manage particular institutional pressures, to protect the reputation of the team and the technique, and it helps ensure that the innovative therapy is not brought to a halt by its failure to live up to overly optimistic anticipations.

## Methods

In this article, we draw upon data collected as part of the Wellcome Trust funded London and Brighton Translational Ethics Centre (LABTEC) research endeavor. This endeavor included a twelve-month ethnographic study of the PMDS, based at a large children’s hospital in the United Kingdom. It is a multidisciplinary service that includes two neurologists, an occupational therapist, two physiotherapists, a speech and language therapist, a specialist nurse, a clinical psychologist, and a clinical research fellow, and a team administrator. It provides DBS to children and young people with severe cases of either primary dystonia (in which dystonia is the only neurological pathology) or secondary dystonia such as dystonic cerebral palsies (in which dystonia coexists alongside other neurological pathologies such as spasticity and contractures).

Interviews were conducted and audio-recorded with each member of the team, and observations of team meetings and interactions with patients were undertaken. Observations were recorded in handwritten notes. Interview transcripts and observation notes were subject to an iterative, thematic analysis using Nvivo coding software. Early on during the fieldwork and the coding of the initial data, it became apparent that team members devoted considerable time and discussion to the management of their patients’ expectations: the researcher (JG) thus made a concerted effort to explore this theme in greater detail during subsequent data collection. As part of this, the researcher observed a goal-setting session, during which PMDS team members attempted to manage the expectations of patients and families by encouraging them to adopt and aim for realistic goals after the DBS system has been implanted. In what follows, we use extracts from interviews to highlight some of the key tensions associated with the management of expectations within the PMDS, and then in order to illustrate aspects of recalibration work, we draw upon observational data from a specific goal-setting session involving several team members, a patient named Carl (pseudonym) and Carl’s mother.

This data collection project was approved by the appropriate NHS Research Ethics Committee. Informed consent was obtained from all participants (clinicians, parents, and patients) sixteen years of age and older, and assent was obtained from all participants under sixteen years of age (consent for their participation was obtained from their parents). Information leaflets for children and young people were modeled on the format provided by Alderson and Morrow (2011).

## Results: Managing Expectations in DBS

### Glamorous Technology: Hope and Truth in DBS

For children and their families, living with dystonia is physically and emotionally challenging. Those with severe forms of the disorder require full-time care and support, and carers often require slings and hoists to move their child. Less-severely affected children and young people who have retained some functional abilities are easily exhausted by having to constantly battle with their involuntary movements, and many feel self-conscious about their appearance.

Both traditional media articles (with titles such as “Deep Brain Stimulation Surgery Provides New Hope for Children,” Wang 2013) and social media have framed DBS as a potentially life-changing therapy for people with dystonia. Children and families, for example, can easily access video recordings of dramatic clinical improvements posted on YouTube. Many of these recordings (which have been posted by patients and their families) contrast striking footage of shaking or rigid involuntary body movement before the DBS implantation with seemingly relaxed and controlled body movement after the implantation. Indeed, much of the journalism and patient-generated social media coverage surrounding DBS for dystonia corresponds to the “regime of hope” identified by Moreira and Palladino (2005).

Clinicians within the PMDS certainly felt that such coverage created unrealistic expectations among the families that came to see them. As the clinical research fellow explained:

There are often misperceptions … the press reports the case studies that do well—so there can be [a] perception that DBS will get [their] child to walk (Interview).

And as the physiotherapist stated, “People often hold hopes and aspirations particularly for something that’s kind of glamorous or technical, something like DBS.” Indeed, during interviews team members often spoke about the way in which families invested hope in DBS. They referred to this as “false hope” and used terms like “misperceptions” and “unrealistic” to frame the initial view held by patients and families and to distinguish it from their own, “realistic” knowledge of what DBS could actually achieve. We can say that the stance of the PMDS clinicians corresponds to a regime of truth (Moreira and Palladino 2005), in which DBS is understood in terms of its most likely impact upon the patient. Such an understanding derives, in large part, from the clinicians’ experiences of previous clinical cases. For all members of the team, it was vital that the “unrealistic expectations” of patients and families were “managed” and realigned with their own understanding of what DBS could achieve. In other words, team members felt it was vital that patients and their families were brought into the regime of truth:

Parents can come with expectations really high. They want the best for their children, and they’re also still probably grieving: the emotions around having a child with long term neuro disability is just unbelievable. It’s huge. And they may come in with unrealistic expectations and sometimes DBS is the only thing out there that they could try for that child. And then you’re having to wind those down … they want their child to talk and [we] are saying we need to rein this down (Physiotherapist Junior).

Sometimes …they’re communicating with the communication aid, they have no speech, and parents are saying, “I want my child to speak.” I will be speaking to them directly about that—that this is actually an unrealistic goal and I’ll talk them through this (Speech and Language Therapist).

The imperative to “rein in” expectations and enroll children and families within a regime of truth was articulated in terms of morality. The clinical team expressed a responsibility to be clear about the limitations of DBS with families, and thus to protect them from the emotional upset and disappointment that would arise if families expected too much from DBS. As one of the physiotherapists suggested, it was “unfair” to “sell DBS to everybody”:

We can potentially bring news [to families] that’s not always easy—being able to be really clear about what we can’t achieve. Because we could sell it to everybody.… But that would be unfair (Physiotherapist).

DBS is highly invasive and it was necessary for members of the team to be clear with families about the limitations of DBS, so that families could make an informed decision about whether or not to proceed with the therapy. Indeed, in an era when facilitating patients’ autonomy and enabling patients’ capacity for decision making are heralded as fundamental to ethical clinical practice, managing the expectations of patients (and bringing them into a regime of truth) can be seen as *an ethical challenge*.

Additionally, the team also felt that encouraging unrealistic expectations, or “false hope,” would be detrimental to the clinical team: “If you paint the most optimistic picture, you are setting yourself up for certain failure” (Neurologist, team meeting). If the clinical outcome did not match the expectations of families, then the reputation of the team could become tarnished and potential patients who could benefit from DBS would be discouraged from approaching the team. Thus, the success of the team, and indeed the success of DBS as a viable therapy for children and young people with dystonia, would be threatened by the “overselling” of DBS.

There is, then, a tension between the exigencies of delivering a novel clinical service, and the hyped, overly optimistic representations of DBS that are portrayed outside of the clinic. As the clinical research fellow states:

We had one child who six weeks after his surgery, was in the press, starting to walk again. And that’s a very worrying thing for us.… Managing the expectations—that can become very difficult.

Thus, as the above examples illustrate, patients arrive at the PMDS having been exposed to a “regime of hope,” and PMDS clinicians believe that an important part of their work is to bring them into a “regime of truth.” In the following section, we explore this aspect of their work.

### Aligning Patients with the Regime of Truth: Recalibration

We refer to the process of bringing children and their families into the regime of truth as *recalibration*. The practice of recalibration requires clinicians to draw on their professional knowledge and clinical experience to foresee how a particular patient may respond to an intervention, and if recalibration is to be successful, communicating this in a manner comprehensible to them. It is, in some respects, an example of what Callon has called *interressement* (Callon 1986), a process whereby an actor attempts to delineate and impose a role or identity on another actor or set of actors. Interressement is a process that generates socio-technical collectives, as an actor brings other actors and entities into a common project by defining, coordinating and aligning their capacities. Similarly, recalibration is a process that involves an actor (the clinician) foreseeing and delineating the likely capacities of another actor (the patient) and imposing this vision on them. It results in the formation of a collective involving the clinician and patient (and often the patient’s family) based upon a shared understanding of a likely future. The resulting calibration of expectations enables clinicians to obtain informed consent from their patients, and in effect it brings patients into the innovation project. In this section, we will explore the process of recalibration as it takes place within the PMDS.

PMDS team members spoke of the need to dispel the rhetoric of “a quick fix” that families tended to associate with DBS and to replace it with a more realistic vision of the future; a future with some incremental gains and possibly some setbacks. As one team member stated:

I genuinely think we are quite good at managing expectations and I think we’re very good at expressing that it will be a long haul, that this is not a fast, rapid sort of change. (Occupational therapist, interview)

This requires a great deal of work from all team members:

The whole team may have an input with [managing expectations], the psychologist [for example] may have to help prepare the child and the family for accepting the likely changes. (Administrator, interview)

For the PMDS team, the key aspect to managing expectations is “goal setting” with children and families. Several weeks before the surgical procedure to implant the DBS hardware, each child and their supporting family member, along with several members of the team, participate in a goal setting session. By this stage, the therapists have conducted a range of presurgical assessments, and they will have discussed the patient’s case with other members of the team. During such team discussions different kinds of information such as official diagnoses, brain scans, and the therapists’ observations from the assessments are brought together, and a collective prediction of how the patient will respond will be made. (While this prediction-making process is not the focus of this article, it is worth noting that it is what Latimer et al. [2006] have described as “micro-political,” during which certain types of evidence and knowledge are foregrounded and prioritized.) During the session, team members use these predictions to negotiate with patients and families a set of goals to aim for once the DBS system has been implanted. These are clearly defined functional goals that ideally pertain to tasks that the child and family feel are important to them, and that team members feel are achievable. These goals are, in other words, very much aligned with the regime of truth; they are informed and guided by the clinicians’ prediction of the *most likely* benefits provided by DBS.

So you have to find out what their hopes are initially. You have to then really agree what’s a realistic expectation.… (Clinical Research Fellow, interview)

It’s nit-picking out those functional goals that you feel could be targeted.… We [establish] more functional goals to make it easier [for example] for the care giver: seating tolerance … standing to get dressed. (Junior Physiotherapist, interview)

These goals do not entail massive or dramatic leaps of recovery (even though a few patients do indeed experience seemingly dramatic improvements). Rather, they entail modest improvements in the patient’s ability to perform specific tasks:

And what we try to do is set some quite modest goals: “Well what are the five things you’d like to improve?” So is it dressing, is it how you sit in a chair? And [we’re] saying, “Okay well how satisfied would you be if you could do that? And how important is that to you?” (Clinical Research Fellow, interview)

Thus, it is via the establishment of these clearly defined goals that team members attempt to realign the “unrealistic” hopes of families with their own “realistic” knowledge of how the patient is most likely to respond.

Goal setting with children and their families involves the construction of an imagined future in notable contrast to those overly optimistic portrayals of DBS that circulate in the media (cf. Gilbert and Ovadia 2011). The futures constructed during the goal-setting session are highly specific and tailored to each child and their family, and are tainted with uncertainty. In the process, patients and their supporting family members are encouraged to rationally reflect on their current limitations and their hopes for the future.

In order to illustrate this in greater detail, we will now turn to a specific goal-setting session involving Carl, a sixteen-year-old patient with secondary dystonia, Carl’s mother, and several members of the team including the occupational therapist, one of the physiotherapists and the clinical psychologist. Here we will see a specific example of recalibration as team members attempt to align Carl and his mother within a regime of truth.

In the session, team members elucidate the tasks that Carl would ideally like to perform. Inevitably, this involves asking patients to envisage their future:

OT: Okay, lets think. You are going to be seventeen—what are the sorts of things you would like to do as a seventeen-year-old?

C: I want a job!

OT: And what stops you now?

Mm: He is not able to use public transport on his own, and I have to motivate him in the mornings, make sure he eats, gets dressed, showers.

Team members then use these tasks as the basis for establishing realistic goals with the patient. This involves honing in on specific tasks, prompting Carl or his mother to clearly articulate the cause of the problem:

OT: You wanted to use public transport?

C: Yes.

OT: Is this a problem because you are worried that you will attract attention or is it because you cannot physically manage?

Mm: If he has one of his ticks, it can be very difficult to get on a bus. They throw him off his feet.

It is at this point, once the cause of the hindrance has been clarified, that the team members will draw upon their earlier observations of the patient to provide an opinion as to how DBS may benefit them. In Carl’s case, much of his instability is from muscle weakness rather than dystonia, and DBS is unlikely to directly improve his ability to use public transport.

PT: We noticed you have muscle weakness around your pelvis that DBS won’t improve … I don’t think we should put this down as a goal for DBS.… I think you will always find a bus, a moving platform, hard.

Here we can see how the team conveys their “realistic” expectations to Carl by drawing on specific aspects of his day-to-day life. In the process of doing this, they actively construct or forecast an expected future, which in Carl’s case is a future where using public transport will still be difficult. As the session continues, the imagined future acquires more specific detail:

PT: Okay, so what would you like to do?

C: Go to town with my friends.… It is the staggering; I want to be able to walk down the High Street with my friends.

Psy: We need to clarify: What would it take to improve your confidence? Would it be not falling at all? Or falling less?

C: Just less falls and less jerky movements.

Psy: So, would just a little bit of improvement, then, help with your confidence, do you think?

C: Yes.

Psy: Because some people might not be happy if they still had some visible signs of the movement disorder. It is good that you think a little improvement would help with your confidence.

Here, Carl is being prompted by the team to envisage a personalized future where DBS provides small improvements, but where he still has visible signs of the movement disorder. It is a modest future in which DBS does not provide dramatic or remarkable benefits. Here is another example:

C: I would like to be able to handwrite on a clear page without making a mark all over the page.

OT: I think your computer is your best option. DBS can maybe help you a bit, but you won’t be able to rely on your handwriting.

And another example:

PT: Carl, tell me about shaving. Why does mum do it for you?

Carl: It pulls on my hair, it is really sore.

OT: His arm pulls away and the hair gets caught in the shaver. It is definitely the involuntary movements that are making it difficult to shave.… If DBS does reduce your involuntary movements … you will find it easier.

Here we see the vision that is being constructed is also tainted with uncertainty highlighted by the phrase “*If* DBS is able to reduce your involuntary movements.…” Indeed, this point is emphasized in the following extract from the end of Carl’s session, particularly in the PT’s reference to her “gut feeling” about Carl’s response to DBS.

PT: Improvements with secondary dystonia are more modest. Many people are happy regardless, they are happy that they gave it a go. Also, it is clear that many people have higher expectations than they tend to let on. That is why we are going over all these things so carefully, and why we are documenting all this. Our gut feeling is that you should expect modest gains. We won’t remove your movement disorder, but we think we will reduce the severity and quantity of your jerky movements.

Thus, by the end of the session the participants have collectively constructed a modest, uncertain, highly personal future for Carl. Carl has been encouraged to imagine a future in which, after having the DBS system implanted, he will still have problems using public transport, he will have visible signs of his movement disorder, he will have to continue to rely on his computer, and where it may be easier for him to shave. Carl, in other words, has been encouraged to re-imagine his future in accordance with a regime of truth, as understood by the PMDS team members. Here, then, we have seen the specific elements of work involved in recalibration within the PMDS: team members prompt the patient to articulate his hopes in specific terms, and by utilizing their professional knowledge and clinical experience, draw on these terms to communicate a personalized vision of the future. The result of this is the establishment of a common understanding of the patient’s likely future, and thus, the patient and supporting family members are brought into a “regime of truth.”

### Difficulties with Managing Expectations: Undercurrents of Hope

The experiences of the PMDS team suggest that recalibration is challenging work, particularly when it pertains to an innovative clinical intervention. Clearly foreseeing a patient’s likely clinical response is an obvious difficulty. With a novel therapy such as DBS for dystonia, there is little in the way of established clinical knowledge that can be drawn upon to make a prediction. As one of the team members stated:

The only way to really find out how you’re going to do is to do it, and that’s a big challenge. So you can give guidance from our experience and try and say what are realistic expectations, but actually I think we’re still at that stage where we can only be very crude with that. And it’s very difficult for us to give anything more than that. (Clinical Research Fellow, interview)

In addition to this uncertainty are differences of opinion on the effectiveness of DBS:

There [are] differences within the team about how team members might think someone is likely to benefit. There are team members who are more optimistic and more positive than others. And I think it can be a difficult thing. (Clinical Research Fellow, interview)

This lack of established knowledge is particularly problematic given that the clinicians feel pressured to be as clear as possible with patients. As a team member states:

[We] have to be very clear if the child is not going to walk or is not going to talk or is not going to be able to independently do this, that and the other. That obviously has to be made very clear. And that can be quite tricky. (Team Administrator, interview)

Recalibration, then, involves carefully managing the tension between uncertainty and the imperative to be clear with patients. As we saw with the example of Carl earlier, the PMDS clinicians manage this tension by using phrases that highlight the provisionality of their assessment, such as “our gut feeling is,” “*if* DBS is able to …,” and “DBS can maybe help you a bit.”

Recalibration work can also be very difficult for patients and families. Unsurprisingly, the process of moving from within a “regime of hope” to a “regime of truth” can be emotionally challenging for patients, and PMDS team members spoke of the “sense of loss” (physiotherapist) experienced by families as they realized that the likely impact of DBS was not what they had initially hoped for. The clinical psychologist spoke of this as “shocking the patient”:

I know that the doctors and everyone else in the team are always really as clear as they can … almost to the point of shocking a patient and saying, you know, “This could happen, this could happen.” (Clinical Psychologist)

Yet despite the “shocking” experience for patients and their families, recalibration does not necessarily entail purging all “unrealistic” expectations. Recalibration involves aligning patients and families within a regime of truth, but an undercurrent of hope may remain. The PMDS clinicians were well aware of this:

Really we all know secretly that whatever we say and whatever the parents agree, when you stimulate them, they all want their child to be the one that starts walking. (Clinical Research Fellow, interview)

I think deep down, everybody, even after doing the goals, deep down, I think loads of people expect more. (Occupational Therapist, interview)

The goal setting session is everyone’s most important session … it really makes the reality of what’s happening … [but] I used to see when I went over to the surgeries [during which the DBS hardware is implanted], and the kids would wake up and then they’d start moving again and you can see it in their parents’ faces—they’re expecting them to be still, even though you’ve gone over and over and over it’s not going to happen. You can see that they were hoping that it would be a miracle (Nurse, interview).

Team members stated that if they sensed that patients and their families had too much hope, then DBS would not be offered to them. In such cases, recalibration has failed: the clinicians have failed to align the expectations of the patient or patient’s family with their own expectations. The occupational therapist describes such a case:

We had a young girl … she was quite a functional, delightful girl, she could do so much. And the dad was like, “I want her to be normal because otherwise I can’t give her to get married,” … And then that started to worry [us], because … when you got into more detail he really wanted a miracle … we didn’t put her through DBS because we didn’t feel that the goals were realistic and we explained [this] to the family.

This specific example also hints at the importance of recalibration in the process of medical innovation. The adoption of DBS as a therapy for children and young people with dystonia within clinical settings depends upon the capacity of clinicians to align patients and families with the regime of truth—the imperative to secure informed consent and the pressure to protect their reputation will discourage clinicians such as the PMDS team from offering a novel therapy to those children or families who have immutably high hopes.

## Discussion: Recalibration and Innovation Work

The development and dissemination of DBS exemplify important tensions associated with biomedicine in contemporary society (Gardner 2013). Like many novel and highly technical biomedical developments, DBS has been the source of considerable hype and hope, and these optimistic portrayals and promissory futures have provided momentum to the dissemination of the DBS technique. Yet, as we have shown in this article, the dissemination of DBS also entails the construction of less hopeful and uncertain futures. The pioneering clinicians integrating DBS as a therapy for children and young people with dystonia must carefully manage the expectations of their patients; they must carefully construct personalized, modest, and uncertain futures during their day-to-day clinical work with patients, and enroll them within this vision of the future. If this cannot be done to the satisfaction of team members, then the therapy will be withheld.

By drawing attention to the construction of these modest and uncertain futures, this article contributes to the emerging work in the “sociology of low expectations” (Fitzgerald 2014; Tutton 2011; Pickersgill 2011). This body of work has shown that innovative biomedical projects are characterized by intertwined discourses of hope, pessimism, and ambivalence, and that this complex entanglement of registers provides actors with a useful intellectual language to pursue and work within biomedical projects, particularly those that are precarious such as neurobiological autism research (Fitzgerald 2014). Innovation projects may be animated by the construction of optimistic futures in the present time, but much of this work is propelled by actors with visions of the future that can be modest, uncertain, and ambivalent. However, while this work—like much of the work with the sociology of expectations—has focused on the constructed futures associated with “early stage” innovations, this article has explored the way in which futures are constructed and mobilized at the “late stage” of biomedical innovation; that is, the adoption stage, where a novel healthcare technique (DBS) has been translated into a clinical therapy that sits somewhere between experimental therapy and routine clinical therapy. Using DBS as a case study, we have illustrated that the pioneering clinicians working at this “late stage” of innovation also deploy visions of the future that are modest, uncertain, and highly personalized, and which are in notable tension with the more promissory visions of the future that circulate in the public domain. Indeed, we suggest that the construction and communication of these futures to prospective patients—what we have called *recalibration*—is a vital part of the work that is done in the late-stage of innovation. The tension between hyped portrayals of a biomedical intervention and the exigencies of clinical practice (such as the imperative to obtain informed consent) necessitates labor that adjusts the expectations of patients. Recalibration is a process through which this occurs. We have framed this recalibration work as a process through which actors attempt to pull patients from a “regime of hope,” in which action is orientated by the anticipation of a “high reward” such as a miraculous clinical outcome, into a “regime of truth” in which action is oriented according to knowledge of “what is most likely to be achieved.”

There are, as we demonstrated with the PMDS goal-setting session, two key aspects to recalibration: First, clinician(s) draw on their professional knowledge and clinical expertise to foresee how the patient is most likely to respond to the therapy, and second, they communicate this vision of the future to the child and family in an attempt to readjust their expectations. We have also illustrated that recalibration is challenging work. It is necessary for clinicians to be as clear as possible with patients, but the novelty of the therapy makes it difficult to anticipate the likely clinical outcome. Patients, too, may find it very emotionally challenging to accept the future that is being presented to them, and even if they do accept it, an undercurrent of hope for a miraculous clinical outcome often remains. The form that recalibration work takes will no doubt vary between clinical settings. The PMDS, for example, is situated within a context that facilitates multidisciplinary service provision: the hospital within which they are based was specifically designed to foster interdisciplinary care, and the NHS tariff system for pediatric services supports multidisciplinary teams. Consequently, recalibration within the PMDS is a multidisciplinary affair, drawing on the skill-set and expertise of professions from several different backgrounds. While the goal-setting session-method of recalibration used by PMDS clinicians might be unique to them, we suggest that the key aspects of this work (foreseeing the likely clinical outcome and communicating this to patients) are an important part of the clinical labor that takes place in all contexts where clinicians are providing novel therapies based upon innovative technologies or techniques. We also suggest that, just as hype and over-optimistic representations provide necessary momentum to innovation projects in their early stage, the construction of “lower” expectations may be necessary for the eventual stabilization of many innovations, particularly clinical innovations concerned with the management of chronic illnesses.

We suggest that recalibration can be seen as a process of enrolling the patient within an innovation alliance. As various science and technology studies theorists have argued, biomedical innovations emerge from, and are shaped by, socio-technical networks that can include researchers and clinicians, industry, regulators, and patient-support groups (Brown and Webster 2004). The success of a biomedical project depends upon the ability of proponents to build and expand socio-technical networks by enrolling other actors through appealing to their interests and goals (often via hype), or as Latour has argued (Latour 1987, 113-15), by “reshuffling” the goals of others who consequently feel compelled to join the network. Recalibration is a process that expands a socio-technical network (or a biomedical project) to include patients, and it does this by “reshuffling” the interests and goals of patients so that they align with what is perceived by clinicians to be “achievable.”

More precisely, recalibration encourages patients and families to adopt future-orientated dispositions that are conducive to technological innovation projects in an era characterized by the valorization of self-determination and self-responsibility. Studies on the “political economy of hope” have illustrated that the management and appropriation of hope is implicated in a form of biological citizenship, in which empowered citizens actively engage with researchers and clinicians, share patient experiences, and make use of the media (Novas 2006; Rose and Novas 2005; Brown 2015). Hope, then, can provide a dynamism for particular a form of individualistic, self-determining, and self-responsible biopolitics (Petersen and Wilkinson 2014). What we see with recalibration is an attempt to discipline and channel this dynamism into specific socio-technical projects. A comparison between recalibration and the oncology hope scales studied by Brown (2015) is useful here. Hope scales, Brown notes, attempt to render a patient’s hope intelligible in such a way that it can become the focus of an intervention. They delineate and quantify “levels” of hope, and they encourage patients to engage in rational reflection. In this way, hope scales function as a disciplinary technology: they encourage the individual to re-orientate themselves toward an “expected,” mandated future based upon diagnostic truth, and to prepare for this future accordingly. Similarly, recalibration is a practice in which a patient’s hopes are rendered intelligible and then subjected to corrective adjustment, and patients and families are thus encouraged to re-orientate themselves toward this mandated future. In this way, patients and their future-orientated dynamism may be co-opted into biomedical innovation projects, and consequently, such projects will be provided with dynamism and will continue, despite their frequent failure to match the hype that often surround them.
